# Detection and sequencing of porcine circovirus 3 in commercially sourced laboratory mice

**DOI:** 10.1002/vms3.144

**Published:** 2019-02-19

**Authors:** Shouchuan Jiang, Nini Zhou, Yufeng Li, Jiahui An, Tianhao Chang

**Affiliations:** ^1^ Key Laboratory of Bacteriology Ministry of Agriculture College of Veterinary Medicine Nanjing Agricultural University Nanjing China; ^2^ Department of Veterinary Preventive Medicine College of Veterinary Medicine Nanjing Jiangsu China

**Keywords:** genome sequencing, mice, phylogenetic tree, porcine circovirus 3

## Abstract

Porcine circovirus 3 (PCV3), a recently discovered virus, has spread widely in pigs throughout the world. In order to investigate the possibility of mice used to study the infection of PCV3, commercially sourced Balb/C and ICR mice were screened for PCV3 infection. Blood samples were collected from 20 mice (10 each of Balb/c and ICR), DNA was extracted, and subjected to PCR with PCV3 specific primers. We found all 20 serum samples tested positive for PCV3 DNA. From four mice, the complete genomes of PCV3 were amplified and sequenced, and a phylogenetic tree was constructed. The results showed that the amplified genome was 2000 bp, and sequence comparison showed that the homology of the complete genome and ORF2 gene with those of porcine PCV3 are 97.9%–98.8% and 96.9%–98.3%, respectively. Amino acids alignment results showed that the Cap protein of the mouse PCV3 isolates share 90.7%–96.3% amino acid homology with that of the references strains derived from pigs. Phylogenetic analysis based on ORF2 sequences showed that all PCV3 strains clustered together and were clearly separate from other circovirus species. We detected PCV3 in experimental mice in China for the first time, which is an opportunity to use mice to study the infection of PCV3 and a potential hazard to swine industry.

## Introduction

Porcine circoviruses (family *Circoviridae*, genus *Circovirus*) are single‐stranded circular DNA viruses with a non‐enveloped icosahedral capsid. They are among the simplest of viruses, consisting of capsid protein and genome, and are one of the smallest DNA viruses know to infect animals (Palinski *et al*. 2017). To date three porcine circovirus have been described, PCV1, PCV2 and PCV3. PCV1 is non‐pathogenic to pigs, but PCV2 is associated with postweaning multisystemic wasting syndrome (PMWS), porcine dermatitis and nephritis syndrome (PDNS), proliferative necrosis pneumonia (PNP), porcine respiratory syndrome (PRDC), reproductive disorders, congenital tremors, and enteritis. In 2016, a new type of porcine circovirus, PCV3, was identified from dead sows with symptoms like Porcine Dermatitis and Nephropathy Syndrome (Palinski *et al*. 2017). Thereafter, China, South Korea, Poland, Brazil, Italy and other countries have reported PCV3 in swine herds (Faccini *et al*. 2017; Kwon *et al*. 2017; Stadejek *et al*. 2017; Shen *et al*. 2018; Tochetto *et al*. 2018). Sequence analysis has revealed that the genome of PCV3 is 2000 bp and consists of ORF2 and ORF1, which share 55% and 35% homology with that of PCV2 (Palinski *et al*. 2017; Zhang 2017). The PCV3 capsid protein (Cap), is encoded by ORF2 and consists of 214 amino acids (aa) residues (Palinski *et al*. 2017), which is 16–17 aa and 19–20 aa less than the Cap of PCV1 (230–231 aa) and PCV2 (233–234 aa), respectively.

Being mice widely used to study many aspects of PCV2 infection, we set out to test the utility of mice for the study of PCV3 infection. The status of PCV3 in the experimental mice was detected using PCR. We found that the commercially sourced Balb/c and ICR mice were infected by PCV3.

## Materials and methods

### Sample collection and DNA extraction

Blood samples, collected from the orbital vein, were taken from 20 mice. 10 Balb/c mice and 8 ICR mice were purchased from Shanghai SLAC Laboratory Animal Co., Ltd, two ICR mice were purchased from Beijing Vital River Laboratory Animal Technology Co., Ltd. Animal experiments were approved by the Institutional Animal Care and Ethics Committee of Nanjing Agricultural University (Approval No. IACECNAU‐20100902). DNA from 200 *μ*l of serum was extracted using an E.Z.N.A. viral DNA kit (Omega Bio‐tek, Inc) according to manufacturer's instructions. DNA was stored at −20°C for further use.

### PCR amplification of the PCV3 genome

One pair of PCV3 specific primers was used for viral detection and three pairs of primers were used for full‐length genome sequencing (Wen *et al*. 2018) (Table 1). The PCR reactions were carried out in a final volume of 25 *μ*l; each reaction included 12.5 *μ*l 2 × TaqMaster Mix (Vazyme biotech co., inltd.), 2.0 *μ*l of primers, and 2.0 *μ*l template DNA. The PCR reaction was performed in a thermocycler (Eppendorf AG 22331, Hamburg, Germany) as follows: pre‐denaturation at 95°C for 5 min, and then 30 cycles for denaturation at 95°C for 30 s, annealing at 56°C for 30 s, elongation at 72°C for 45–90 s, and finally extension at 72°C for 10 min. PCR products were purified using a Gel Extraction Kit (Bioer Technology Co. Ltd, Hangzhou. China) according to the manufacturer's instructions, then cloned into a pClone007 vector. The recombinant plasmids were sequenced by TSINGKE Biological Technology (Nanjing, Jiangsu, China). DNA sequences were assembled using the ClustalW program of DNAstar.

**Table 1:** Primers used for PCV3 detection and amplification of the full‐length genome

Usage	Primers	Primers sequence (5′‐3′)	PCR product Size (bp)
Detection	PCV3‐F	TCCAAACTTCTTTCGTGCCGTAG	386
	PCV3‐R	GGCTCCAAGACGACCCTTATGC	
	PCV3‐1F	ATTATGGATGCTCCGCACCGTG	553
	PCV3‐1R	CATCTTCTCCGCAACTTCAGTC	
Genome amplification	PCV3‐2F	GACTGAAGTTGCGGAGAAGATG	789
	PCV3‐2R	CATCTTCTCCGCAACTTCAGTC	
	PCV3‐3F	CCCACATGCGAGGGCGTTTACC	895
	PCV3‐3R	CGAGGCCGCTTCATCATCCACT	

### Sequence alignment and phylogenetic analyses

The PCV3 genomes from the four mice (GenBank: MH445393‐MH445396) and reference strains were analyzed using ClustalW from DNAStar to determine nucleotides and amino acids homologies. A phylogenetic tree was constructed using MEGA7.0 software. The maximum likelihood method was used with a bootstrap analysis of 1000 replicates.

## Results

**Figure 1:**
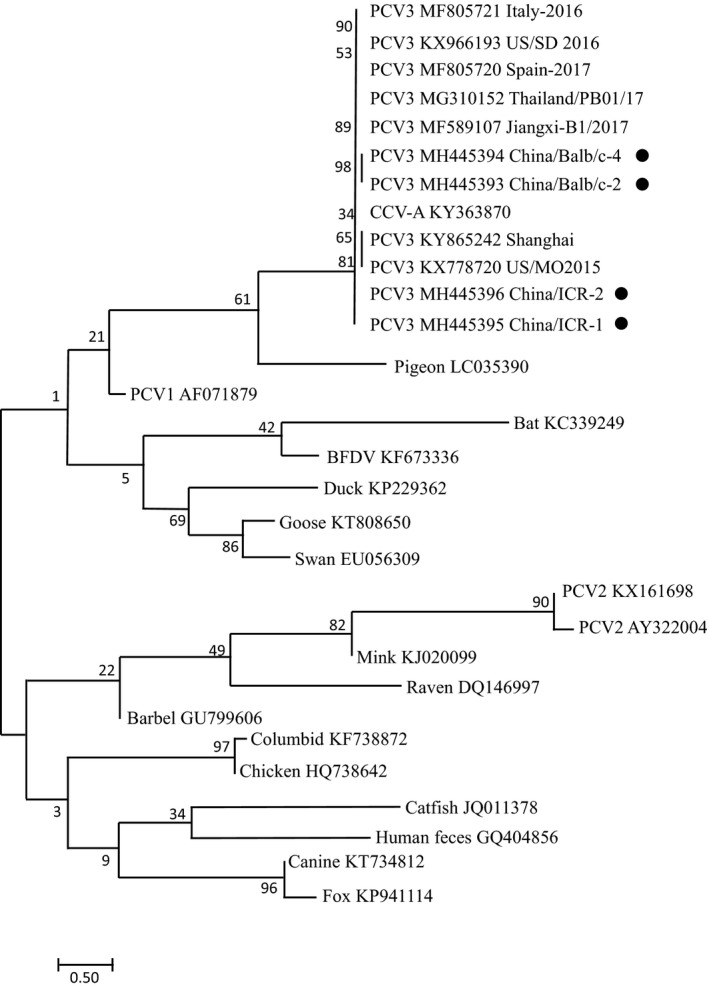
Phylogenetic analysis of the ORF2 of PCV3 derived from mice and reference ORF2 of circovirus species. The tree was constructed using MEGA7.0 software with maximum likelihood method and 1000 replicate sets on bootstrap analysis.

PCR results showed that the serum from all the mice tested was positive for PCV3, The PCV3 genomes from 4 positive samples were cloned and sequenced. Sequence identities within the four full‐length genomes and the ORF2 genes ranged from 98.3% to 99.4% and 96.6%–99.8%, respectively. The full‐length genomes and ORF2 genes share 97.9%–98.8% and 96.9%–98.3% identity, respectively with seven PCV3 strains derived from pigs, and are clearly separated from other circovirus species (Fig. 1) indicating that the genetic relationship between mouse and pig PCV3 is close. Amino acid alignment results showed that the PCV3 Cap proteins from the four mice share 90.7%–96.3% homology with PCV3 Cap proteins from the references strains. As seen in table 2, the Cap proteins from the four mouse PCV3 contained several substitutions compared to the reference sequences. The mouse strains share a common point mutation at position 475 (G475A), resulting in isoleucine being replaced by alanine at position 159. In addition, the strains from Balb/c mice (GenBank MH445393–MH445394) contain point mutations at position 91, 544, 564 598 and 606 (C91T, T544C, G564T, T598A and T606G), amino acids were altered at all positions except 606. Strains from ICR mice (GenBank MH445395–MH445396) contain point mutations at position 118, 143, 244, 250, 353, 355, 426 and 480 (A118G, C143T, A244T, A250C, T353C, A355C, T426C and T480C), amino acids were altered at all positions except 426 and 480. We found point substitutions in ICR mice are higher than that of in Balb/c mice, which is obviously different from that observed in PCV3 derived from pigs. Especially, only 1 common point substitutions were found in ICR mice and MF084994 (source).

## Discussion

Circoviruses are key agents responsible for several syndromes in pigs. Moreover, circoviruses have also been detected in a wide range of species such as birds, dogs, and foxes (Todd 2004; Li *et al*. 2013; Bexton *et al*. 2015). PCV2 is known to spread across species and may cause disease (Li *et al*. 2013; Decaro *et al*. 2014). Phylogenetic analysis of PCV ORF2 sequences reveal that PCV3 has a high degree of phylogentically related with Canine CV (KT734812). In this study, we found that the ORF2 gene of PCV3 sequenced from the commercially sourced lab mice were highly homologous with pig PCV3 strains.

In circoviruses, the capsid protein is the only structural protein, hence it contains the only antigenic epitopes and is the main candidate for the study of PCV2 immunogenicity. However, the Caps between PCV2 and PCV3 share only 30% amino.acid identity, indicating PCV2‐based vaccines may not provide cross‐protection against PCV3 infections in the field. Compared with Cap protein sequence of PCV3 isolated from pigs, some unique aa residue substitutions were identified in the Cap proteins of PCV3 in mice (Table 2).

**Table 2:** Comparison of nucleotides and amino acid substitutions in the Cap protein of the PCV3 strains isolated from four mice, with corresponding sequences of reference strains

Source of strain	Strain	Substitutions (position[nucleotide],amino acid^a^													
		91 (C)	118 (A)	143 (C)	244 (A)	250 (A)	353 (T)	355 (A)	426 (T)	475 (G)	480 (T)	544 (T)	564 (G)	598 (T)	606 (T)
Balb/c mice	MH445393	T								A		C	T	A	G
	MH445394	T								A		C	T	A	G
ICR mice	MH445395		G	T	T	C	C	C	C	A	C				
	MH445396		G	T	T	C	C	C	C	A	C				
Swine	KX778720									A					
	KX966193														
	KY865242														
	MF084994					C									
	MF318451														
	MF859107									A					
	MF805720														
	MF805721														
	MG310152														

a Original amino acid substituted amino acid: Original amino acid (amino acid deletion); substituted amino acid(amino acid addition); NS = no amino acid mutation; R: arginine; N:asparagine; C:cysteine; Q:glutamine; I:isoleucine; L:leucine F:phenylalanine; P:proline; S:sernine; T:threonine; Y:tyrosine V:valine.

We revealed that commercially sourced lab mice from China are infected with PCV3. The experimental Balb/c and ICR mice were propagated in controlled biosafety conditions, where it is unlikely for them to have contact with wild mice. Therefore, it cannot be ruled out that they may have carried this virus when they were domesticated. Further surveillance and studies need to be aimed at identifying the origin of PCV3 in commercial mice and its effect on some biological studies.

## Conclusion

This is the first study to reveal the existence of PCV3 in commercially sourced lab mice. Genome analysis showed that the PCV3 detected from the mice has high similarity with PCV3 derived from pigs. Amino acid alignment reveals some substitutions occurred in the Cap protein when compared with PCV3 derived from pigs. It is a opportunity to use mice to study the infection of PCV3 and a potential hazard to swine industry. Our results reveal mice may play a role in the transmission of PCV3 to pigs.

## Source of funding

This study was supported by the National Key Research and Development Program of China (2016YFD0500701‐3) and Priority Academic Program Development of Jiangsu Higher Education Institutions (PAPD).

## Conflicts of interest

The Authors declare that there is no conflict of interest.

## Contributions

LYF designed and carried out this project. JSC and ZNN contributed in samples collection, data collection and lab works, in a team. JSC analysed data and wrote this article. AJH, and CTH supervised the project and checked the writing of this article.

## Ethics statement

Animal experiments were approved by the Institutional Animal Care and Ethics Committee of Nanjing Agricultural University. (Approval No. IACECNAU20100902).
